# Supplementary material for: Mechanisms governing the pioneering and redistribution capabilities of the non-classical pioneer PU.1
Source: Nat Commun. 2020 Jan 21;11:402. doi: 10.1038/s41467-019-13960-2 (PMC6972792; doi:10.1038/s41467-019-13960-2)
Supplement: Supplementary file 7 — Source data [file 41467_2019_13960_MOESM7_ESM.zip › Source_Data/FigureS2/FigureS2B_MotifScanOutput/KmeansCluster5/homerResults/motif1.similar.html]

motif1

## Information for motif1

A
T
G
C
C
G
T
A
A
T
G
C
C
G
A
T
A
C
G
T
A
G
T
C
A
G
T
C
A
G
C
T
A
G
T
C
G
A
C
T
G
A
C
T
G
A
C
T
  
Reverse Opposite:  

C
T
G
A
C
T
G
A
C
T
G
A
T
C
A
G
T
C
G
A
C
T
A
G
A
C
T
G
C
G
T
A
C
G
T
A
T
A
C
G
G
C
A
T
T
A
C
G
  

|  |  |
| --- | --- |
| p-value: | 1e-2011 |
| log p-value: | -4.631e+03 |
| Information Content per bp: | 1.634 |
| Number of Target Sequences with motif | 3280.0 |
| Percentage of Target Sequences with motif | 82.41% |
| Number of Background Sequences with motif | 4856.2 |
| Percentage of Background Sequences with motif | 11.34% |
| Average Position of motif in Targets | 101.9 +/- 31.7bp |
| Average Position of motif in Background | 102.3 +/- 81.9bp |
| Strand Bias (log2 ratio + to - strand density) | 0.0 |
| Multiplicity (# of sites on avg that occur together) | 1.39 |
| Motif File: | file (matrix) reverse opposite |

### Similar de novo motifs found

|  |  |  |  |  |  |  |  |
| --- | --- | --- | --- | --- | --- | --- | --- |
| Rank | Match Score | Redundant Motif | P-value | log P-value | % of Targets | % of Background | Motif file |
| 1 | 0.978 | A T G C C G T A A T G C C G A T C G A T A G T C A G T C A G C T A G T C G C A T G A C T | 1e-1931 | -4447.583534 | 83.72% | 13.04% | motif file (matrix) |
| 2 | 0.990 | T C G A C T G A C T G A C T G A T C A G T C G A C T A G T A C G G T C A C G T A T A C G G C A T T C A G | 1e-1852 | -4265.746311 | 82.51% | 13.23% | motif file (matrix) |
| 3 | 0.966 | C T G A A T G C C G A T C G A T A G T C A G T C A G C T A G T C G C A T G A C T | 1e-1768 | -4072.240225 | 84.82% | 15.89% | motif file (matrix) |
| 4 | 0.961 | C T G A A T G C G C A T A G C T G A T C G A T C A G C T A G T C G A C T G C A T G A C T | 1e-1630 | -3755.300961 | 84.10% | 17.36% | motif file (matrix) |
| 5 | 0.911 | T C A G C T G A A C T G A C T G C G T A C G T A T A C G G C A T T A C G | 1e-1627 | -3748.624974 | 69.67% | 8.76% | motif file (matrix) |
| 6 | 0.965 | A G T C A G T C G C T A A T G C G C A T G A C T A G T C G A T C A G C T A G T C G A C T G A C T G A C T A G C T | 1e-1600 | -3685.912687 | 79.90% | 14.83% | motif file (matrix) |
| 7 | 0.931 | C T G A A T G C A C G T A C G T A G T C A G T C A G C T A G T C G C A T G C A T G C T A G T C A G A C T | 1e-1531 | -3525.335939 | 87.16% | 21.50% | motif file (matrix) |
| 8 | 0.947 | T C G A A T G C A C G T A C G T A G T C A G T C G A C T A G T C G C A T G C A T G C T A G A C T | 1e-1530 | -3525.140175 | 92.36% | 26.66% | motif file (matrix) |
| 9 | 0.917 | C G T A A T G C A C G T A C G T A G T C A G T C A G C T A G T C | 1e-1527 | -3516.834609 | 83.64% | 18.64% | motif file (matrix) |
| 10 | 0.920 | A G T C C T G A A T G C C G A T C G A T A G T C G A T C G A C T G A C T G A C T | 1e-1423 | -3278.709576 | 79.52% | 17.23% | motif file (matrix) |
| 11 | 0.912 | T C G A A T G C C G A T C G A T T A G C G A T C A G T C G A C T G A C T | 1e-1296 | -2985.924534 | 86.21% | 25.10% | motif file (matrix) |
| 12 | 0.852 | A T G C C G T A A G T C A C G T A C G T A G T C A G T C | 1e-1066 | -2454.797460 | 56.33% | 8.85% | motif file (matrix) |
| 13 | 0.856 | G C A T C T G A A G T C G A T C C G T A A T G C C G A T C G A T T A G C G T A C A G C T | 1e-1051 | -2420.758744 | 60.18% | 11.00% | motif file (matrix) |
| 14 | 0.900 | A T G C A C G T A C G T A G T C A G T C G A C T A T G C G A C T G A C T | 1e-1032 | -2376.384486 | 77.66% | 23.15% | motif file (matrix) |
| 15 | 0.883 | C T G A G C T A C G A T C T A G T G A C T C A G A C T G C G T A G T C A T A C G | 1e-1012 | -2331.172787 | 89.57% | 35.54% | motif file (matrix) |
| 16 | 0.817 | T A C G C T A G A C T G A C T G C G T A C G T A T A C G A G T C | 1e-992 | -2285.041186 | 90.88% | 37.73% | motif file (matrix) |
| 17 | 0.879 | A T G C A C G T A C G T A G T C A G T C A G C T A G T C | 1e-989 | -2277.336309 | 74.95% | 21.87% | motif file (matrix) |
| 18 | 0.845 | G A T C G T C A A T G C C G A T A C G T A G T C G A T C | 1e-902 | -2078.820174 | 72.24% | 21.64% | motif file (matrix) |
| 19 | 0.808 | C T G A A C T G A C G T A C G T A G T C A G T C A G C T | 1e-889 | -2048.339912 | 91.06% | 41.02% | motif file (matrix) |
| 20 | 0.832 | A T C G A C G T A G C T A G T C A G T C A G C T G A T C C G T A G C A T | 1e-729 | -1680.296670 | 47.61% | 9.34% | motif file (matrix) |
| 21 | 0.912 | C A G T T A G C G C T A A T G C A C G T A C G T A G T C A G T C A G C T A G T C G T C A T G C A G C T A C T A G | 1e-703 | -1619.303762 | 35.50% | 4.37% | motif file (matrix) |
| 22 | 0.843 | C T A G G C T A T C A G A C T G C T G A T C G A T A G C G C T A | 1e-641 | -1477.753317 | 69.22% | 25.87% | motif file (matrix) |
| 23 | 0.858 | C T G A C G T A G T C A A G T C T C G A T A C G A T C G G C T A C G T A T A C G | 1e-592 | -1363.245792 | 38.19% | 6.91% | motif file (matrix) |
| 24 | 0.850 | T C A G T C G A G C T A C T G A A C T G A C T G T G C A C G T A C T A G G C A T C T A G C T A G | 1e-535 | -1233.754036 | 32.26% | 5.11% | motif file (matrix) |
| 25 | 0.868 | A T G C A C G T A C G T A G T C A G T C A G C T A G T C G C T A G C T A T G C A C G A T | 1e-355 | -818.013688 | 35.95% | 10.33% | motif file (matrix) |
| 26 | 0.755 | G A T C G A C T A G C T A G T C G A T C G A T C G A T C G A C T A G C T C A G T A T C G T G A C G A T C | 1e-226 | -522.305366 | 21.66% | 5.49% | motif file (matrix) |
| 27 | 0.774 | T C A G T G C A T C G A C T A G C A T G C T A G T C A G T C G A C T G A C T A G G T A C G C T A | 1e-189 | -436.162928 | 19.42% | 5.20% | motif file (matrix) |
| 28 | 0.767 | T C G A C T G A C T A G C T A G C T A G T C A G C T G A C T G A T C A G T A G C G T C A T C A G C T A G T A G C | 1e-159 | -366.450377 | 11.58% | 2.10% | motif file (matrix) |
| 29 | 0.754 | C T G A A T C G A C G T A C G T A G T C G A T C A G T C C G A T C A G T G C A T A G C T G A C T G A C T | 1e-148 | -342.939211 | 11.61% | 2.28% | motif file (matrix) |
| 30 | 0.778 | G C T A T C G A A G T C C G A T C A G T A T G C A T G C A C G T A C T G G C A T | 1e-145 | -335.928301 | 14.65% | 3.77% | motif file (matrix) |
| 31 | 0.823 | G A T C G T C A C G T A C T A G C T A G C T A G C T A G C T A G C G T A C G T A T C G A C A G T A T G C A G T C | 1e-142 | -327.076162 | 6.21% | 0.47% | motif file (matrix) |
| 32 | 0.779 | A C T G A T G C A C G T A C G T A G T C A G T C A G C T A G T C G A C T A G C T A C G T A G T C G A T C G A T C | 1e-108 | -248.849011 | 9.05% | 1.90% | motif file (matrix) |
| 33 | 0.769 | A T G C G C T A T A G C G C A T C G A T G A T C G T A C C G A T C G A T A C G T G C A T A G C T C A G T | 1e-107 | -247.456710 | 4.15% | 0.23% | motif file (matrix) |
| 34 | 0.776 | T G A C T G C A A T G C G C A T C G A T A T G C A T G C G A C T C G A T C A T G A C G T G C A T | 1e-104 | -240.556639 | 6.96% | 1.10% | motif file (matrix) |
